# “Does Institutional Quality, Natural Resources, Globalization, and Renewable Energy Contribute to Environmental Pollution in China? Role of Financialization”

**DOI:** 10.3389/fpubh.2022.849946

**Published:** 2022-03-31

**Authors:** Waqar Ameer, Azka Amin, Helian Xu

**Affiliations:** ^1^School of Economics, Shandong Technology and Business University, Yantai, China; ^2^School of Economics and Trade, Hunan University, Changsha, China; ^3^Department of Business Administration, Sukkur IBA University, Sukkur, Pakistan

**Keywords:** financial development, CO_2_ emissions, institutional quality, renewable energy, sustainability development

## Abstract

Our study explores the impact of financialization on carbon emissions by utilizing diverse financialization proxies, particularly for China. We examine the impact of financialization, institutional quality, globalization, natural resources, trade openness, and renewable and nonrenewable energy consumption on environmental pollution over the period 1996–2017 by utilizing dynamic autoregressive distributed lag (ARDL) simulations. The empirical findings of the study indicate that institutional quality, trade, globalization, natural resources, and renewable energy consumption significantly decrease environmental pollution in the long run, while foreign direct investment and financialization have neutral effects on carbon emissions. Our findings demonstrate that a 1% increase in institutional quality, trade, IFDI, renewable energy, and globalization leads to a decrease in CO2 emissions by 0.198, 0.016, 0.075, 0.010, and 0.072%, respectively. Even though financialization indexes contributed insignificantly to environmental degradation, other explanatory variables significantly affected carbon emissions through indirect effects of financialization. Financialization indexes behave in a similar context, and these proxy indicators are good parameters to understand the complex nature of financialization. Moreover, in order to achieve low carbon emissions and sustainable development, countries need viable financial institutions that focus on green growth by promoting clean production process strategies to ensure the reduction of CO_2_ emissions.

## Introduction

Global warming has emerged as a challenging environmental issue in recent decades (1–4) and it may terribly affect the health of human beings (5–8). Over the last two decades, environmental degradation has become a serious challenge worldwide that has forced researchers, scholars, and policy makers to think properly about environmental issues and provide environmentally friendly policies (6–8). Such a rise in the worldwide temperature due to global warming and its harmful effects on the environment caused the arrangement of a United Nations convention on climate change in 1992. Later, the Kyoto Protocol convention was arranged in 1997 and subsequently, the Paris Contract session was convened in 2015 to reduce global warming by imposing environmental restrictions on gas emissions. Environmental degradation arises due to emissions of greenhouse gases and thus, it has become a serious issue worldwide in environmental disputes (9). Carbon dioxide (CO_2_) emits approximately 75% of greenhouse gases (10). The world's top 10 carbon-emitting countries represent almost 67% of the total emissions of the world, and China is included among the top 10 carbon-emitting countries of the world (2). Accordingly, strict disciplinary measures are needed to reduce CO_2_ emissions from top pollution-emitting countries.

China has undergone marvelous economic development since it opened its borders for international capital flows in recent decades. Hence, this rapid economic development of the Chinese economy severely affected the environment and gave rise to environmental pollution. A lot of empirical studies have investigated the various determinants of environmental pollution particularly in the case of China (11, 12). Industrial development is highly associated with environmental issues and empirical evidence confirms that a large number of industries emit huge amounts of greenhouse gases in China (13). At the present time, there is roughly 50% of annual greenhouse gas emission growth in China and it must be urgently evaluated by environmental protection regulations or must be regulated by the Chinese laws of environmental sustainable development. China started the policy to develop environmental institutions in 1972 and the Chinese government sent an official delegation to attend the United Nation conference on environmental development and sustainability in 1972 which was held in Stockholm^1^. Local environmental regulations were enforced in China in 1989 when the environmental protection law was approved by the National People's Congress of People's Republic of China. Up until now, 29 laws have been formulated by the National People's Congress and its standing committee of P.R. China regarding environmental protection and sustainable development. China's first political priority is to develop a pollution-free environment. Recently in China, large scale environmental policies have been implemented at the national level, such as the 2015 Environmental Protection Law, 2017 Environmental Protection Tax Law, and 2018 Environmental Protection Tax Law (14).

The Chinese government is persistently working hard to minimize the harmful effects of industrial development on environmental sustainability as per reports of different 5-year plans (FYPs). Economic development and sustainable environment is the core agenda of China (15). The Chinese supreme legislature body approved an environmental resolution on climate change within the context of environmental challenges in 2009 (16). The International Energy Agency (IEA) presented a critical analysis report and confirmed that the Chinese government has significantly launched environmental protection policy measures at national, provincial, and lower district levels in order to attain the targets of green energy and sustainable environment (17). The Chinese government has the realized harmful effects of gas emissions from inefficient power plants in its 5-year plans and strictly enforced environmentally friendly policies to reduce carbon emissions and environmental pollution. The FYPs are developed by the Chinese government, and environment protection strategies were the top priority of government officials, scholars, and environmental experts in the different 5-year plans of China (18). The environmental protection laws (EPLs) of China highlighted environmental challenges and provided effective environmental planning and effective legislative and institutional settings in the context of the environmental governance system of China. The EPLs are designed to deal with environmental challenges, highlighting the shortcomings in the environmental system, and propose better solutions for a sustainable environment (19).

China has achieved a lot of economic development in the recent decades but, alternatively, this rapid economic development adversely affected the environment and raised a lot of environmental challenges and issues for China (20). As per the latest statistical reports, China has become the world's largest CO_2_ emitter and energy consumer (21). Its energy consumption per unit of GDP is twice the world's average and per capita CO_2_ emissions have increased by 40% of world's average. The growth rate of CO_2_ emissions in China has risen more than 11 percent per annum (22). Accordingly, this rapid increase of CO_2_ emissions in the last couple of years might result in environmental degradation of the Chinese economy (23, 24). Empirical literature has keenly emphasized the significance of the relationship between environmental pollution and economic growth (25–28). The economic rationality and validity of the environmental Kuznets curve (EKC) hypothesis is widely debated while exploring the environment-growth nexus (29–35) and energy-environment-growth nexus in the long run and short run (7, 36, 37).

Some empirical studies, such as Boutabba (38), Bekhet et al. (39), Gokmenoglu and Sadeghieh, (40), Wang et al. (41), and Shahbaz et al. (42), considered financial development as a key explanatory variable, and empirical findings of these studies strongly supported the fact that financial development determines changes in carbon emissions. The recent literature has focused on investigating the impact of financialization on environmental development (38). Different scholars have diverse opinions on the nexus between financial development and environmental degradation. A lot of empirical studies conclude that financial development aggravates environmental degradation (41, 43–48). A stable financial system not only increases the efficiency of the financial sector but also contributes toward rapid economic development of a country (44, 49–51). The development of the stock markets and financial institutions reduces financing expenses and eases the liquidity requirements of firms, thus it help firms to invest in new projects to expand production, stimulate energy demand and thus, in turn, can give rise to environmental pollution (52–54). Financial markets encourage the public to borrow loans from financial institutions and buy heavy weight vehicles that can be the principal cause of the increase in carbon emissions (44).

Conversely, other schools of thought argue that financial development reduces environmental pollution by employing energy-efficient technology (20, 50, 55–63). With advancement of technology, financial markets developed globally and turned in to multinational corporations. Financial markets promote investment activities and monitor performance of the companies. The principal role of the financial sector is to bring lenders and borrowers close to each other with the purpose of efficient utilization of capital in a profitable way. Financial markets play a key role in the economy development of a country (64). Financial institutions especially banks and stock exchanges utilize public deposit funds and savings for investment purposes in a productive way and thus, in turn, contribute toward economic development of a country (65).

The prime contribution of this empirical study is to familiarize readers with the diverse financialization proxies which are introduced by IMF in order to understand and explore more comprehensively the impact of financialization indexes on carbon emissions particularly in the case of China. A large number of previous empirical studies have used a single proxy to denote financial development. For example, a study of Al-Mulali et al. (30) focused on the European region and investigated the impact of financial development on carbon emissions. They employed domestic credit to the private sector (% GDP) proxy variable to denote financial development. There are many empirical studies, such as Ahmed (66) and Haseeb et al. (67) for BRIC countries; Ali et al. (68) for Nigeria; and Kayani et al. (69) for emitter countries, which have employed a simple and single proxy variable to denote financial development. These empirical studies found a positive impact of financial development on environmental degradation. On the other hand, using the same proxy variable to denote financialization, some empirical studies, such as Jalil and Feridun (20) for China; Shahbaz et al. (70) for South Africa; Shahbaz et al. (71) for Malaysia; Nasreen and Anwar (72) for low-, middle-, and high-income countries; Lee et al. (73) for OECD; Abbasi and Riaz (10) for Pakistan; Dogan and Seker (74) for top renewable energy countries; and Gill et al. (75) for Malaysia, concluded that financial development unfavorably and negatively affects environmental issues. Henceforth by employing the same proxy variable to denote financialization, some empirical studies, such as Ozturk and Acaravci (76) for Turkey; and Seetanah et al. (9) for Small Island Developing States, found an insignificant impact of financial liberalization on environmental degradation in the long run.

Many empirical studies, such as Ziaei (77) for European, East Asian, and Oceania countries; Ali et al. (68) for Nigeria; and Jiang and Ma (78) for developed, emerging, and developing countries, have used an alternative simple proxy variable (domestic credit provided by financial sector) to investigate its impact on environmental issues. Additionally, some of the recent empirical studies applied several other proxy variables to denote financial development for robustness checks. For example, Tsaurai (79) employed three different proxies of financial development in his analysis for Africa. These three different financialization proxy measures employed are broad money, domestic credit to financial sector, and domestic credit to private sector by banks. Katircioglu and Taşpinar (80) used four different proxies, such as liquid liabilities; broad money supply; domestic credits to the banking and private sector; and ratio of commercial bank assets to central bank assets plus commercial bank assets, and they found an adverse impact of financial liberalization on carbon emissions for Turkey. Shoaib et al. (51) employed five different proxies, such as stock market capitalization; domestic credit to private sector; stock market turnover ratio; bank z-score; and bank net interest margin, to investigate the impact of financial development on environmental pollution for developed and developing countries and found a favorable impact of financial development on carbon emissions.

The fact that the above mentioned empirical studies utilized more than one proxy for financial liberalization index is quite motivational because these indicators of financialization may not comprehensively cover the complex nature of financialization (65). In order to overcome the limitation of single proxies of financial liberalization, our study fills this gap by employing multiple or diverse indices of financial development (65), thus to the best to our knowledge, this is the first study that extends the literature by utilizing multiple or different indices of financialization particularly in the case of China. These indices summarize the state of financial markets and institution in terms of depth^2^, access^3^, and efficiency^4^ (81, 82). The diversity of the financial structure proposes multiple indicators to measure the effects of financial development across countries (2). Amin et al. (2) tried to explore the impact of financialization on carbon emissions by utilizing a comprehensive index of nine different financial index proxies for the top 10 carbon-emitting^5^ countries based on panel data studies. However, it is widely recognized that any potential inference drawn from these cross-country studies provides only a general understanding of the linkage between the variables, and thus are unable to offer much guidance on policy implications for each country (23). Hence, the focus of this research is to investigate the impact of financialization on carbon emissions by employing multiple or different financialization proxies^6^, particularly in the case of China.

A few empirical research studies, such as Xiong and Qi (84), Jalil and Feridun (20), and Zhang (50), explored the impact of financial development on carbon emissions by employing simple proxy variables for financialization, particularly in the case of China, but we extend the contribution of these empirical studies by introducing multiple indexes of financialization proxies in order to explore more comprehensively the impact of financialization on carbon emissions, particularly in the case of China. Xiong and Qi (84) provided valuable insight between financial development and carbon emissions by incorporating interesting variables in the model but this study was narrowly focused at the Chinese provincial level and also employed a single proxy for the financialization index. Accordingly, we are conceptualizing key variables such as institutional quality, globalization, FDI inflows, and natural resources in order to reduce the problems of omitted variable bias in our proposed study. These variables are significantly ignored in the prior works of Zhang (50), and Jalil and Feridun (20) because empirical study of Tamazian and Rao (57) confirm the role of improved governance in reducing environmental problems. Equally, empirical literature, such as Frankel and Romer (85), Bhattacharya et al. (86), Sharif et al. (87), also confirm the significant role of FDI inflows, natural resources, and renewable energy in reducing environmental problems. Moreover, our analysis is robust as we are utilizing an updated dataset for the rapidly growing economy of China from 1996 to 2017 annually, and also we are applying the most robust dynamic autoregressive distributed lag (ARDL)^7^ methods to control the endogeneity, multicollinearity, and autocorrelations issues for the time series dataset of our empirical research study and thus this advanced methodology is not applied in the prior works of Xiong and Qi (84), Jalil and Feridun (20), and Zhang (50).

Our study contributes to the existing literature in several ways. Firstly, it introduces financialization by utilizing various proxies that have never been utilized before for China. Secondly, the impact of institutional quality has been ignored in earlier literature, our study identified the impact of institutions on environmental degradation. Thirdly, we have employed an up-to-date econometric methodology.

The remainder of the study is as follows. Section Model, Data, and Econometric Methodology defines the model, data, and methods; Section Results and Discussion explores the empirical results and discussion; and Section Conclusion and Policy Recommendations presents the conclusion of the study and policy implications.

## Model, Data, and Econometric Methodology

This study investigates the role of institutional quality, financialization indexes, FDI, natural resources, trade openness, globalization, and renewable and non-renewable energy consumption on carbon emissions for China from 1996 to 2017 annually. Inspired by the work of Al-Mulali et al. (30), we have extended our model by adding some important variables.


(1) $$\begin{array}{l} {\text{CO}}_{2\text{t}}=\beta_{1}+\beta_{2}{\text{ GOV}}_{t}+\beta_{3}{\text{ FD Index}}_{t}+\beta_{4}{\text{ TRADE}}_{t}+\beta_{5}{\text{ IFDI}}_{t}\\ \;+\beta_{6}{\text{ RENRGY}}_{t}+\beta_{7}{\text{ NONRENRGY}}_{t}+\beta_{8}{\text{NRSOURCES}}_{t}\\ \;+\beta_{9}{\text{ GLOBAL}}_{t}+\varepsilon_{t} \end{array}$$

where CO^2^ emission is an environmental indicator; GOV is the governance index, FD Index is the financial development index, TRADE is trade openness, IFDI is a foreign direct investment inflow, RENRGY is renewable energy consumption, NONRENRGY is non-renewable energy consumption, NRSOURCES is natural resources, and GLOBAL is the globalization index. The description of all indicators is reported in Table 1. The summary statistics of all the variables are reported in Table 2 which demonstrates the mean, maximum, minimum, and standard deviation values of the variables. The results of descriptive statistics depict positive trends for all the variables. These variations seem sufficient for further empirical estimation.

**Table 1:** Variables description.

**Abbreviations**	**Variable name**	**Definition and scale of measurement**	**Source**
CO_2_	Carbon dioxide emissions	Metric tons	WDI, World Bank https://databank.worldbank.org/source/world-development-indicators
GOV	GOV stands for institutional quality index	GOV is extracted by applying principal component (PCA) methods. GOV is an aggregated index of six individual governance indicators (rule of law; control of corruption; regulatory quality; government effectiveness; political stability and no violence; voice and accountability)	WGI, World Bank https://databank.worldbank.org/source/worldwide-governance-indicators
FD Index	Financial development index	FD Index stands for different proxies of the financialization index, such as financial development index (FDI), financial institutional index (FII), financial markets index (FMI), and financial markets depth index (FMDI). These proxy indexes are utilized to measure the diverse nature of financial development and these multiple financialization proxies are introduced by the International Monetary Fund (IMF)	IMF website https://www.imf.org/en/Data
TRADE	Trade openness	(% GDP)	WDI, World Bank https://databank.worldbank.org/source/world-development-indicators
IFDI	Foreign direct investment inflows	(% GDP)	WDI, World Bank https://databank.worldbank.org/source/world-development-indicators
RENRGY	Renewable energy consumption	(% of total final energy consumption)	WDI, World Bank https://databank.worldbank.org/source/world-development-indicators
NONRENRGY	Non-renewable energy consumption	(% of total final energy consumption)	WDI, World Bank https://databank.worldbank.org/source/world-development-indicators
NRSOURCES	Natural resources	Coal	WDI, World Bank https://databank.worldbank.org/source/world-development-indicators
GLOBAL	Globalization	Globalization calculated in indexes	WDI, World Bank https://databank.worldbank.org/source/world-development-indicators

**Table 2:** Summary statistics.

**Variable**	**Obs**	**Mean**	**Std. dev**.	**Min**	**Max**
CO2	22	4.91	1.92	2.51	7.32
GOV	22	0.000	1.43	−2.69	2.12
FDIX	22	0.4545	0.5096	0	1
FIIX	22	0.1818	0.3947	0	1
FMIX	22	0.5454	0.5096	0	1
FMDIX	22	0.4545	0.5096	0	1
TRADE	22	46.06	10.22	32.42	64.47
IFDI	22	3.46	0.965	1.34	4.72
RENRGY	22	19.20	7.75	11.33	30.53
NONRENRGY	22	84.77	3.80	78.93	88.89
NRSOURCES	22	1.22	1.250	0.067	4.83
Global	22	84.65	1.44	81.4	86.6

As per the results in Table 2, the mean value of carbon emissions is 4.91 and its range starts from the minimum value of 2.51 and ends with the maximum value of 7.32. Financialization index proxies (FDIX, FIIX, FMIX, and FMDIX) assume an average value of 0.4545 (FDIX), 0.1818 (FIIX), 0.5454 (FMIX), and 0.4545 (FMDIX) with the minimum value of these proxy indexes starting from a value of 0 and up to a maximum value of 1. The average value of the governance index (GOV) is zero, the GOV index range starts from a minimum value of 0 and ends with a maximum value of 1. The mean values of FDI inflows (IFDI) and TRADE are 3.46 and 46.06, respectively. The average values of renewable energy (RENRGY) and nonrenewable energy (NONRENRGY) are 19.20 and 84.77, respectively. The average values of globalization index and natural resources are 84.65 and 1.22, respectively.

### Econometric Methodology

Jordan and Philips (88) developed a new dynamic stimulated ARDL method namely the dynamic ARDL simulations approach to overcome the complications in short- and long-run examinations of the original ARDL approach. The dynamic simulations ARDL approach estimates and predicts the probability change of the regression and on one regressor, while keeping other regressors unchanged. On the other hand, the Pesaran ARDL approach only examines the long-run and short-run linkage between variables. Although the implementation of the ARDL approach is very convenient, its dynamic form accepts the first difference and multiple lags of both regressor and regression (88). To estimate the dynamic ARDL simulations, all the variables in the econometric model must be stationary at the first difference I(I), and there should be cointegration among all indicators (37, 88). This method uses multivariate normal distribution to simulate the vector of parameters 5,000 times. The equational form of the dynamic ARDL simulations approach is presented in equation (1).


(2) $$\begin{array}{l} \Delta y_{t}=\emptyset_{0}Y_{t-1}+\emptyset_{1}{\left(X_{1}\right)}_{t-1}+\ldots +\emptyset_{k}{\left(X_{k}\right)}_{t-1}\\ \;+\sum_{k=1}^{m}\sigma_{i}\Delta {\left(y\right)}_{t-1}+\sum_{l=0}^{n_{i}}\partial_{ij}\Delta {\left(x_{1}\right)}_{t-j}+\ldots .\\ \;+\sum_{l=0}^{n_{k}}\partial_{kj}\Delta {\left(x_{k}\right)}_{t-j}+\mu_{t} \end{array}$$

In equation (1), y demonstrates the variation in the dependent variable; ∅_0_ is the intercept; t-1 is the maximum p-value of the regressor; *n*_*k*_shows the number of lags; Δ is the first difference; t is the time period, while μ is the error term. The null hypothesis of no cointegration *H*_0_ = ∅_0_ + ∅_1_ + … + ∅_*k*_ = 0 is checked against the alternate hypothesis *H*_*A*_ = ∅_0_ + ∅_1_ + … + ∅_*k*_ ≠ 0. The null hypothesis of no co-integration is rejected if the calculated value of F-statistics is greater than its critical value.


(3) $$\begin{array}{l} \Delta \ln{\left({\text{CO}}_{2}\right)}_{it}=\beta_{0}\Delta \ln{\left({\text{CO}}_{2}\right)}_{\text{it}-1}+\alpha_{1}\Delta \ln{\left(\text{GOV}\right)}_{\text{it}}\\ \;+\;\delta_{1}\Delta \ln{\left(\text{GOV}\right)}_{\text{it}-1}+\alpha_{2}\Delta \ln{\left(\text{FD}\right)}_{\text{it}}\\ \;+\;\delta_{2}\Delta \ln{\left(\text{FD}\right)}_{\text{it}-1}\;+\;\alpha_{3}\;\Delta \ln(\text{TRADE})\text{it}\\ \;+\delta_{3}\;\Delta \ln{\left(\text{TRADE}\right)}_{\text{it}-1}\;+\alpha_{4}\;\Delta \ln{\left(\text{IFDI}\right)}_{\text{it}}\;\\ \;+\;\delta_{4\;}\Delta \ln{\left(\text{IFDI}\right)}_{\text{it}-1}\;+\alpha_{5}\;\Delta \ln{\left(\text{RENRGY}\right)}_{\text{it}}\;\\ \;+\delta_{5}\;\Delta \ln{\left(\text{RENRGY}\right)}_{\text{it}-1}+\alpha_{6}\;\Delta \ln{\left(\text{NONRENRGY}\right)}_{\text{it}}\;\\ \begin{array}{l} \;+\;\delta_{6}\;\Delta \ln{\left(\text{NONRENRGY}\right)}_{\text{it}-1}\\ \;+\alpha_{7}\;\Delta \ln{\left(\text{NRSOURCES}\right)}_{\text{it}} \end{array}\\ \;+\delta_{7}\;\Delta \ln{\left(\text{NRSOURCES}\right)}_{\text{it}-1}+\alpha_{8}\;\Delta \ln{\left(\text{GLOBAL}\right)}_{\text{it}}\\ \;+\delta_{8}\;\Delta \ln{\left(\text{GLOBAL}\right)}_{\text{it}-1}+\;\varepsilon \text{it} \end{array}$$

The novelty of our study is that it employs the dynamic ARDL approach based on dynamic simulations which has recently been added to the existing literature by Sarkodie et al. (37).

## Results and Discussion

Before applying the dynamic ARDL simulations approach, the first step is to check the stationarity of all variables, that is, the dependent variable should be stationary at first difference I(1), while all independent variables must be stationary at level or at the first difference, i.e., I(0) or I(1).

This study applies augmented Dickey-Fuller (ADF) and Phillip-Perron (PP) unit root tests to check the stationarity of all variables. The results of unit root tests in Table 3 demonstrate that all variables are stationary at first difference I(1).

**Table 3:** Unit root test results.

	**ADF**		**Phillips–Peron**	
**Variable**	**level**	**First difference**	**level**	**First difference**
CO2	−1.221	−2.434^***^	2.423^**^	−1.235^**^
GOV	−1.220	−2.646^***^	−1.996^**^	−4.464^***^
IFDI	−0.173	−2.499^***^	−1.544	−4.631^***^
TRADE	−2.058^**^	−1.667^**^	−0.173^*^	−3.128^***^
RENRGY	−1.833^**^	−1.628^**^	−2.622^**^	−1.331^*^
NONRENRGY	−2.183^**^	−1.778^**^	1.887	−1.519^*^
FDIX	−0.386	−5.119^***^	−0.354	−10.231^***^
FIIX	−0.243	−3.082^***^	0.000	−4.359^***^
FMIX	−1.065	−5.119^***^	0.000	−4.359
FMDIX	−0.386	−2.646^***^	−0.354	−10.231^***^
GLOBAL	−1.851^**^	−2.114^**^	1.991	−5.138^***^
NRSOURCES	−1.225	−2.831^***^	−1.346	−7.888^***^

*, **, and *** *represent 1%, 5%, and 10%, respectively*.

### Dynamic ARDL Simulations

The results of the dynamic ARDL simulations are reported in Table 4. The governance has a negative relationship with CO_2_ emissions which implies that an increase in the quality of institutional quality leads to a decrease in CO_2_ emissions in China. Our empirical estimations are consistent with the findings of Tamazian and Rao (57) which supported the role of improved governance in reducing environmental problems. Establishment of stable financial, economic, and environmental institutions contributes to green energy, thus helping to mitigate environmental degradation. It can be said that the expansion of government spending and development of institutional quality stimulate economic activities in an economy, attract foreign direct investment and trade, which ultimately strengthens the scale effects on carbon emissions.

**Table 4:** Results for dynamic ARDL simulations.

**Regressors**	**Model 1**	**Model 2**	**Model 3**	**Model 4**
Lagged CO2	−0.5456^**^ (−3.77)	−0.4530^**^ (−4.05)	−0.2155 (−1.79)	−0.5456^**^ (−3.77)
GOV	−0.1988^**^ (−5.00)	−0.0804 (−1.42)	−0.1542^**^ (−4.03)	−0.1988^**^ (−5.00)
Δ GOV	0.1439 (2.44)	0.00087 (0.03)	−0.0312 (−2.39)	−0.0401 (−2.64)
FDIX	0.3453 (2.06)			
Δ FDIX	0.1439 (2.44)			
FIIX		−0.1630 (−1.53)		
Δ FIIX		−0.13683 (−1.31)		
FMIX			0.4166 (2.90)	
Δ FMIX			0.2705 (2.64)	
FMDIX				0.3453 (2.06)
Δ FMDIX				0.1439 (2.44)
TRADE	−0.01648^**^ (−3.84)	−0.02074^**^ (−5.13)	−0.0162^**^, −5.39	−0.0164^**^ (−3.84)
Δ TRADE	0.0018 (0.320)	−0.0017 (−0.25)	0.0116 (2.70)	0.0018 (0.32)
IFDI	−0.0759 (−2.26)	−0.03410 (−0.88)	−0.0595, −2.06	−0.0759 (−2.26)
Δ IFDI	0.0788* (3.44)	0.0806 (2.79)	0.1188^**^ (5.54)	0.0788* (3.44)
RENRGY	−0.1017^**^ (−5.21)	−0.0976^*^, −3.19	−0.0656, −2.47	−0.1017^**^ (−5.21)
Δ RENRGY	0.0759 (2.07)	0.0044 (0.12)	0.0182 (0.46)	0.0759 (2.07)
NONRENRGY	0.1210 (1.75)	0.1088 (1.90)	0.0138 (0.35)	0.1210 (1.75)
Δ NONRENRGY	0.3819^**^ (5.29)	0.2338^*^ (3.09)	0.3370^**^ (4.76)	0.3819^**^ (5.29)
NRSOURCES	0.1722 (2.70)	0.0603 (0.75)	0.2413^**^ (4.20)	0.1722 (2.70)
Δ NRSOURCES	0.0688 1.59	0.0246 (0.61)	0.0867^*^ (3.19)	0.0688, 1.59
GLOBAL	−0.0728 (−3.12)	−0.0367, −1.27	−0.09018^**^, −4.02	−0.0728^*^, −3.12
Δ GLOBAL	0.0512 (2.21)	0.0064 0.25	0.0372 (1.56)	0.0512, 2.21
CONS	1.396 (0.27)	−0.7771, −0.12	9.348 2.37	1.396, 0.27
Breusch–Godfrey LM	0.2016	0.1442	0.2376	0.2016
Breusch–Pagan (heteroscedasticity)	0.3995	0.3995	0.3995	0.3995
Skewness and Kurtosis (normality)	0.3288, 0.2629	0.0387, 0.9344	0.3512, 0.2378	0.3288, 0.2629

***, **, and * *denote 1, 5, and 10% levels of significance. **Δ** denotes the value of the coefficient of the explanatory variables in the short run. T-values are in parenthesis ()*.

The negative and significant relationship between trade and CO_2_ emissions implies that international trade helps to mitigate environmental degradation. A potential reason is that China's higher economic growth rate and increased income have reduced trade barriers, which ultimately leads to improve environmental quality. Furthermore, China has improved its manufacturing structure. Due to the increased demand for traded goods, low-polluting goods produced in China have greatly contributed to the reduction of CO_2_ emissions. Our findings are consistent with Chen et al. (89), Yazdi and Beygi (90), Hao and Liu (91), and Shahbaz et al. (70).

The coefficient of foreign direct investment (IFDI) shows insignificant results in the long run while it depicts a positive and significant relationship in the short run. The negative coefficient of renewable energy consumption (RENRGY) demonstrates that a rise in the share of renewable energy consumption adversely affects CO_2_ emissions in China. In China, with increasing concerns regarding health environmental costs of CO_2_ emissions, RENRGY must become an effective substitute for fossil fuels (such as oil, coal, and natural gas). Our results are similar to those of Anwar et al. (92), Wang et al. (41), and Bekun et al. (93), who found that an increase in the demand of energy and enormous consumption of non-renewable energy sources exerts an adverse impact on the environment.

The impact of natural resources on CO_2_ emissions is positive and significant for China. Abundant natural resources minimize the need for fossil fuel energy; in addition, these results are related to the use of China's own energy sources (such as natural gas and renewable energy), which emit fewer emissions than fossil energy sources. The coefficient of globalization shows a negative and significant relationship with CO2 emissions in China. Shahbaz et al. (94) argued that globalization adversely affects CO2 emissions through income effect, scale effect, and technique effect. In addition, this also confirms the Chinese government's willingness and concern to reduce carbon dioxide emissions by adopting environmental policies with rapid economic growth.

Empirical results show that financialization indexes^8^ (FDIX, FMIX, and FMDI) positively but insignificantly contribute toward environmental pollution except FIIX^9^. Conversely, FIIX reduces environmental pollution but the effects are insignificant. Even though financialization indexes contributed insignificantly to environmental degradation when we analyze the separate impact of each financialization index on carbon emissions in model 1, model 2, model 3, and model 4, other explanatory variables^10^ significantly affected carbon emissions in all models through indirect effects of these financial indicators. We notice that financial indexes affected carbon emissions indirectly through other explanatory variables in model 1, model 2, model 3, and model 4 as all of the explanatory variables significantly affected carbon emissions in the models of our proposed study. As per estimations, we notice that financialization development indexes are quite similar in nature and behave in a similar context, and these proxy indexes can be utilized to measure the diverse nature of financial development. Our empirical estimations are parallel to those by Seetanah et al. (9) and Ozturk and Acaravci (76) who found neutral effects of financialization on environmental degradation in the long run.

The results of the diagnostic tests are presented in Table 4. The diagnostic tests are applied to check the consistency of econometric models. The results of the Breusch-Godfrey LM test demonstrate that no serial correlation was found in the model. The results of Breusch-Pagan show the absence of heteroscedasticity in the model. To check the normality of the dataset, we have applied skewness and kurtosis tests. The results demonstrate that normal distribution existed under the null hypothesis.

## Conclusion and Policy Recommendations

In recent decades, global warming has emerged as a challenging issue that may cause deterioration of sustainable development across the globe. Over the last couple of decades, CO_2_ emissions have widely and significantly contributed to global warming which ultimately heinously affects climate change and increases environmental pollution across the globe. Accordingly, it is quite interesting to explore those factors which widely contributed to carbon emissions and environmental pollution. This empirical study explores the impact of diverse financial development indexes, institutional quality, trade, globalization, natural resources, and renewable and nonrenewable energy consumption on carbon emissions for China over the period 1996–2017 annually.

This empirical study has applied advanced methodology; namely, dynamic time series ARDL simulations proposed by (88). The dynamic ARDL simulations overcome limitations in the already existing ARDL approach model. This approach used 5000 simulations of the vector of parameters by utilizing multivariate normal distribution. The study examined the impact of financialization indexes, institutional quality, globalization, natural resources, and various other environmental factors, for instance, renewable and nonrenewable energy consumption, foreign direct investment, and trade on environmental degradation. Empirical results conclude that institutional quality, globalization, natural resources, trade, and renewable energy consumption significantly and negatively contributed toward carbon emissions, while foreign direct investment and nonrenewable energy consumption had neutral effects on CO_2_ emissions. Even though financialization indexes contributed insignificantly to environmental degradation, other explanatory variables significantly affected carbon emission in all models through indirect effects of these financial indicators.

Financialization indexes affected carbon emissions indirectly through other explanatory variables in model 1, model 2, model 3, and model 4. We infer from our empirical results that financialization indexes are quite similar in nature and behave in a similar context, and these proxy indicators are good parameters to analyze the diverse nature of financialization. Based on our empirical results, this study provides some important policy implications. Firstly, institutional quality significantly decreases carbon emissions, thus, researchers must formulate strong policies to strengthen financial and local institutions in order to significantly reduce environmental pollution. The lack of environmental protection policies in financial institutions has led to increased CO_2_ emissions. Therefore, it is recommended to strengthen financial institutions and adopt environmentally friendly policies to decrease CO_2_ emissions. The establishment of stable financial, economic, and environmental institutions contributes to green energy, thus helping to mitigate environmental degradation. The findings of the study demonstrate that globalization, natural resources, trade, and renewable energy consumption contribute toward the reduction of environmental pollution in China which promotes sustainable development. In order to promote an eco-friendly environment, policy makers and public institutions should follow global environmentally friendly laws and promote a globalized business environment in order to significantly attract foreign companies and global investors; thus, subsequently, more globalized environmental laws and environmentally friendly policies can significantly reduce environmental issues. In order to maintain a high-quality environment, policy makers must establish consistency between environment and economic policies through utilization of natural resources and globalization. Our findings demonstrate that a 1% increase in institutional quality, trade, IFDI, renewable energy, and globalization leads to a decrease in CO2 emissions by 0.198, 0.016, 0.075, 0.010, and 0.072%, respectively.

As far as the limitations of the current study are concerned, we had to remove some financialization proxies due to omitted variable bias or model misspecification error. Henceforth, we can further extend this study by adding all of the nine diverse indexes of financialization proxies^11^ in order to comprehensively understand the role of financialization in terms of environmental degradation, particularly for China. Additionally, the dataset of our empirical study comprises information from 1996 to 2017 annually, which is another limitation of our study. Thus, we can further extend our study by overcoming these limitations by utilizing all nine indexes of financialization proxies and using updated datasets (conditionally depends upon the availability of datasets) in order to derive more interesting policy implications particularly in the case of China. Additionally, this is a single country analysis, we suggest that future studies can be extended to a larger sample of countries in order to obtain broader conclusions.

## Data Availability Statement

The original contributions presented in the study are included in the article/supplementary materials, further inquiries can be directed to the corresponding authors.

## Author Contributions

All authors listed have made a substantial, direct, and intellectual contribution to the work and approved it for publication.

## Funding

This project was supported by National Social Science Fund of China (Grant No. 21BJY113).

## Conflict of Interest

The authors declare that the research was conducted in the absence of any commercial or financial relationships that could be construed as a potential conflict of interest.

## Publisher's Note

All claims expressed in this article are solely those of the authors and do not necessarily represent those of their affiliated organizations, or those of the publisher, the editors and the reviewers. Any product that may be evaluated in this article, or claim that may be made by its manufacturer, is not guaranteed or endorsed by the publisher.

## Footnotes

^1^https://www.cfr.org/backgrounder/chinas-environmental-crisis (accessed on 10 July 2018).

^2^Size and liquidity.

^3^Ability of enterprises to obtain financial services.

^4^Ability of institutions to provide their financial services at the lowest possible cost with optimum returns.

^5^China is also included among the top 10 carbon-emitting countries.

^6^We had to remove some proxies from the financialization index due to omitted variable bias in the model; these novel financialization proxies were introduced by (83).

^7^Dynamic autoregressive distribution lag simulations methodology.

^8^Financial development index (FDIX); financial market index (FMIX); financial markets depth index (FMDIX).

^9^Financial institutional index (FIIX).

^10^Institutional quality; natural resources; trade liberalization; globalization; renewable energy.

^11^These diverse indexes of nine financialization proxies are introduced by International Monetary Fund (IMF).
